# Proteomic analysis of dental pulp from deciduous teeth in comparison to permanent teeth: an in-vitro study

**DOI:** 10.1007/s40368-025-01043-4

**Published:** 2025-04-18

**Authors:** S. Horsophonphong, S. Roytrakul, K. Lertruangpanya, N. Kitkumthorn, R. Surarit

**Affiliations:** 1 Department of Pediatric Dentistry, Faculty of Dentistry, Mahidol University, 6 Yothi Road, Ratchathewi, Bangkok, Thailand; 2 Functional Proteomics Technology Laboratory, National Center for Genetic Engineering and Biotechnology, National Science and Technology Development Agency, Pathumthani, Thailand; 3 School of Dentistry, Mae Fah Luang University, Chiang Rai, Thailand; 4 Department of Oral Biology, Faculty of Dentistry, Mahidol University, Bangkok, Thailand; 5 Faculty of Dentistry, Siam University, Bangkok, Thailand

**Keywords:** Dental pulp, Proteomics, Mass spectrometry, Deciduous dentition, Permanent dentition

## Abstract

**Purpose:**

The aims of this study were to identify proteomic profiles of dental pulp from deciduous teeth and compare the profiles of the two dentitions.

**Methods:**

Teeth that were caries-free and had normal pulp conditions were collected from twelve healthy individuals. The obtained teeth consisted of deciduous teeth (*n* = 6) and permanent teeth (*n* = 6). Proteins were extracted from pulp tissue and then analysed using liquid chromatography-tandem mass spectrometry. MaxQuant was used to identify and quantify proteins from raw mass spectrometry data of the collected deciduous and previously analysed permanent dental pulp. Differentially expressed proteins (DEPs) between the dental pulp of the two dentitions were identified by a statistical analysis conducted using Metaboanalyst with criteria *P*-value < 0.05 and fold change > 2.

**Results:**

A total of 3,636 proteins were identified in the dental pulp of deciduous teeth. The biological process functional classifications of these proteins were primarily concerned with cellular process, biological regulation, metabolic process and response to stimulus. Dental pulp protein profiles differed significantly between deciduous and permanent teeth, with 736 proteins being differentially expressed, the majority of which were highly expressed in the pulp of deciduous teeth. Pathway analysis indicated DEPs to be involved in tumour necrosis factor (TNF) signalling, nuclear factor kappa B signalling, and odontoclast/osteoclast differentiation.

**Conclusion:**

While the dental pulp of deciduous and permanent teeth shares some characteristics, there are also significant differences in protein expression, with the TNF signalling pathway and odontoclast/osteoclast differentiation being promoted in the dental pulp of deciduous teeth.

**Supplementary Information:**

The online version contains supplementary material available at 10.1007/s40368-025-01043-4.

## Introduction

Humans have two sets of dentition: deciduous and permanent. Deciduous teeth are the first teeth to emerge into the oral cavity from six to thirty-three months after birth and remain the main set of teeth during childhood (Holt et al. 2000). Although deciduous teeth are eventually replaced by permanent teeth, they are essential for a child's masticatory function, phonetics, esthetics and occlusion (Castro et al. 2017).

Deciduous and permanent teeth share many similarities. In general, both dentitions appear to have similar functions and comparable composition (Sheid and Weiss 2012). Both teeth consist of hard and soft tissues. Enamel, dentin and cementum are the hard tissues that make up both teeth, while dental pulp, which is found inside the tooth, is the soft tissue (Schaffner and Lussi 2019). One of the most notable distinctions between the two dentitions is the physiologic root resorption, a natural process that occurs only in deciduous teeth (Arnold et al. 2019). The process of physiologic root resorption begins shortly after the root of the deciduous tooth is fully formed and is a series of events that occur gradually throughout the tooth's life, rather than a single event that occurs right before the tooth is exfoliated (Harokopakis-Hajishengallis 2007; Sheid and Weiss 2012). Physiologic resorption of the tooth involves the resorption of cementum, dentin and the dental pulp. (Arnold et al. 2019; Harokopakis-Hajishengallis 2007)

Dental pulp, the soft tissue of the tooth, is comprised of odontoblasts, fibroblasts, pulp mesenchymal stem cells, immune cells, lymphatic vessels and nerve and blood supplies (Pohl et al. 2024). Dental pulp plays an important role in tooth development, functions, defence mechanisms and response to various stimuli (Arnold et al. 2019; Pohl et al. 2024; Schaffner and Lussi 2019). In short, it is essential for the well-being of the teeth. The pulp of the two dentitions reportedly differs in terms of cellular composition, responses to stimuli and pulp tissue resorption (Arnold et al. 2019; Bardellini et al. 2016; Nukaeow et al. 2022). For instance, the odontoblastic layer was thinner in deciduous teeth compared to permanent teeth (Arnold et al. 2019). The reparative activity of odontoblasts in deciduous teeth was shown to be lower than in permanent teeth (Bardellini et al. 2016). More importantly, pulp resorption as a physiologic process can only be seen in deciduous teeth, while pulp resorption in permanent teeth is the result of a pathological process (Arnold et al. 2019; Harokopakis-Hajishengallis 2007). Furthermore, the pulp responses to certain therapeutic techniques varied between the two dentitions. For example, direct pulp capping and pulpotomy treated with calcium hydroxide in deciduous teeth resulted in unpredictable outcomes and a significant failure from internal resorption (Canoğlu et al. 2022; Jha et al. 2021; Moretti et al. 2008). While, in permanent teeth, the same medicament is recommended as the standard for vital pulp therapy due to the success of the treatment outcomes (Akhlaghi and Khademi 2015; Ricucci et al. 2023).

There are several differences between the dental pulp of deciduous and permanent teeth, however, our current understanding and literature on this subject are limited and incomplete. One of the methods for investigating how an organ works and functions is an analysis of its proteins (Morris et al. 2022). Proteomics is the large-scale investigation of protein functions, interactions, cellular activities, structures and compositions so as to gain a greater comprehension of tissue or organ biology (Chandramouli and Qian 2009). So far, the literature has only examined dental pulp proteomes for permanent dentition (Eckhard et al. 2015; Eckhardt et al. 2014; Feridouni Khamaneh et al. 2021; Loureiro et al. 2020; Silva et al. 2021), with no investigation yet into the pulp proteomes of deciduous teeth. Due to the fact that the dental pulp of the two dentitions is known to be distinct, it can be expected that their proteomes also differ. Therefore, we aimed to examine protein profiles of dental pulp from deciduous teeth and to compare these profiles with pulp from permanent teeth.

## Methods

### Sample selection

This research was approved by the Ethical Institutional Review Board, Faculty of Dentistry and the Faculty of Pharmacy, Mahidol University (COE.No.MU-DT/PY-IRB2024/004.2802). Dental pulp of deciduous teeth was collected from six donors meeting the inclusion criteria of healthy children under the age of twelve and not taking any medications. The teeth were caries-free with normal pulp conditions and had at least two-thirds of their root length remaining. The indication for extraction was prolonged retention or for orthodontic purposes. Patients and legal guardians provided informed consent prior to extraction and collection of the teeth. For permanent teeth, dental pulp tissue was collected from caries-free third molars of six healthy adults (three males and three females), ranging in age from 20 to 25 years, which was reported and analysed in a previous study by Lertruangpanya et al. (2024).

### Sample collection and storage

**Fig. 1:**
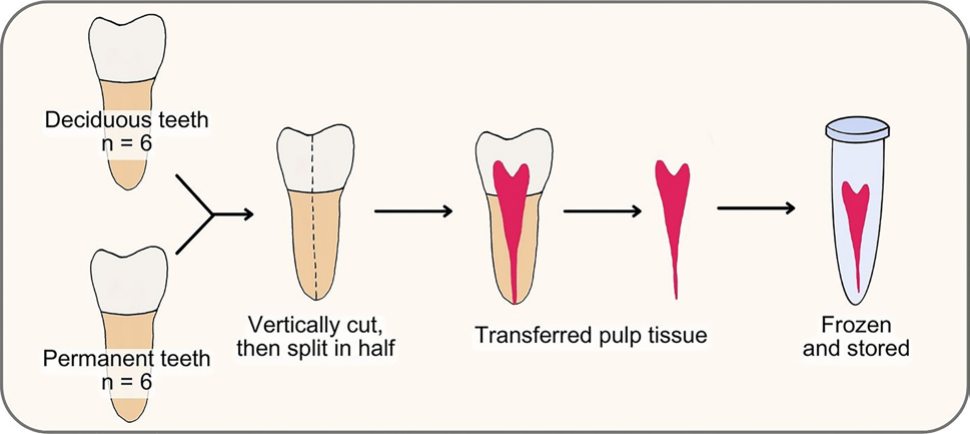
Flow diagram of sample collection, dental pulp tissue extraction and preservation

Following extraction, the outside surfaces of the teeth were rinsed and cleaned with normal saline, and the remaining soft tissue attached to the tooth surface was mechanically removed using a periodontal curette. The teeth were vertically cut with a water-cooled tapered diamond bur, then split in half with an elevator to extract the pulp tissue. Dental pulp tissue was transferred on ice, then snap-frozen in liquid nitrogen and stored at -80 °C. A diagram of sample collection, pulp tissue obtaining and storage is illustrated in Fig. 1.

### Protein extraction and quantification

**Fig. 2:**
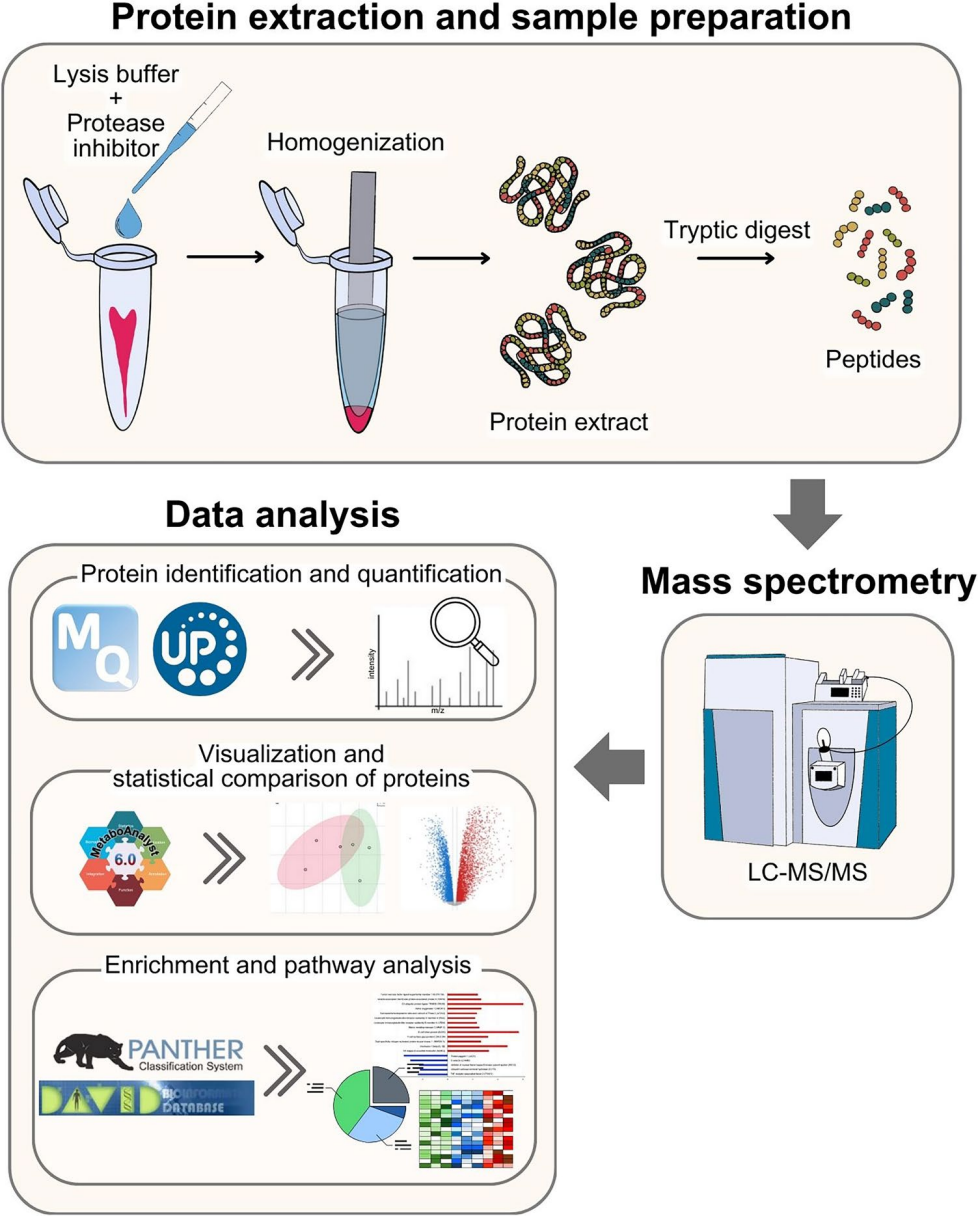
Flow diagram of sample preparation, sample processing, mass spectrometry and proteomic data analysis

A previously published protocol was used to extract and quantify dental pulp protein (Lertruangpanya et al. 2024). In short, pulp tissues were added to a mixture of protein lysis buffer (Sigma-Aldrich, St. Louis, MO, USA) and protease inhibitor (Roche, Mannheim, Germany) at a concentration of 10:1 and manually homogenised using a tissue homogeniser (Fig. 2). Following that, the samples were vortexed and centrifuged for 30 minutes. The supernatant was retrieved, and total protein was quantified using the microplate bicinchoninic acid protein assay kit (Thermo Scientific™, Waltham, MA, USA) by mixing the samples with working reagent, incubating at 37 °C for 30 minutes, and measuring absorbance at 562 nm with a microplate reader (BioTek, Winooski, VT, USA). The protein concentration was determined based on a standard curve of bovine serum albumin (Smith et al. 1985).

### Mass spectrometry

Five micrograms of protein were dissolved in 10 mM ammonium bicarbonate for in-solution digestion. Disulfide bonds were reduced, and sulfhydryl groups were alkylated at room temperature and kept in darkness. The proteins were then broken down using sequencing-grade porcine trypsin. The resultant peptides were first dried and then dissolved in 0.1% formic acid. The resultant peptides of each pulp tissue sample were individually processed and separately injected into an Ultimate3000 Nano/Capillary LC System (Thermo Scientific, UK) equipped with a ZenoTOF 7600 mass spectrometer (SCIEX, Framingham, MA, USA) in triplicate (Fig. 2) (Lertruangpanya et al. 2024; Songjang et al. 2023).

### Data analysis and comparison of pulp proteomes between deciduous and permanent teeth

The raw mass-spectrometric data of proteins obtained from the dental pulp of deciduous teeth in this study have been deposited in the jPOST Repository at https://jpostdb.org under accession number PDX052858 (Okuda et al. 2017). Equivalent raw data for permanent teeth dental pulp were obtained from data previously deposited in the jPOST Repository (accession: PDX050860) (Lertruangpanya et al. 2024). Both datasets were processed using MaxQuant (version 2.4.2.0) to identify and quantify proteins. The Andromeda search engine was used to align Tandem mass spectrometry (MS/MS) spectra with the UniProt *Homo sapiens* database (Fig. 2). Analysis parameters, including limits on missed cleavages, mass tolerance and modifications, were as previously described by Lertruangpanya et al. (2024). Protein identification required peptides of at least seven amino acids and at least one unique peptide. Proteins were identified if they had at least two peptides, one of which was unique, with a 1% false discovery rate (Lertruangpanya et al. 2024; Tyanova et al. 2016).

MaxQuant ProteinGroups.txt files were imported into MetaboAnalyst 6.0 for visualisation and statistical comparison of proteins between the two groups (Pang et al. 2024). Differentially expressed proteins (DEPs) were identified as those with a fold change greater than 2.0 and a *P*-value < 0.05. Gene Ontology (GO) enrichment analysis for biological process, cellular component, and molecular function terms was performed using the Panther online database (Mi et al. 2019). Kyoto Encyclopedia of Genes and Genomes (KEGG) pathway enrichment analysis was conducted using the David online database (Sherman et al. 2022). The databases and tools used for statistical and bioinformatic analysis of proteomic data are illustrated in Fig. 2.

## Results

**Fig. 3:**
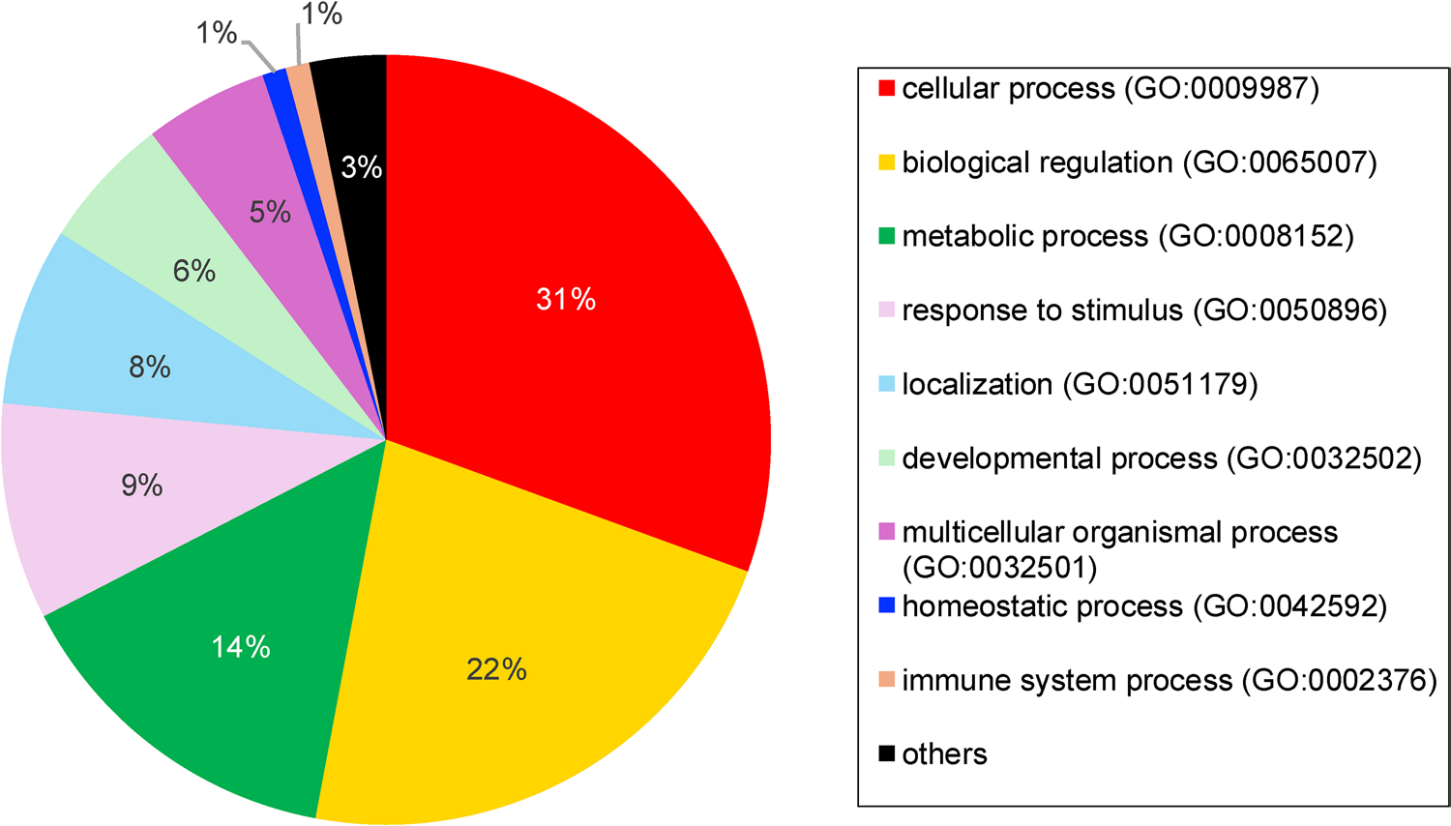
Functional classification of proteins found in deciduous teeth dental pulp according to GO biological process terms

**Fig. 4:**
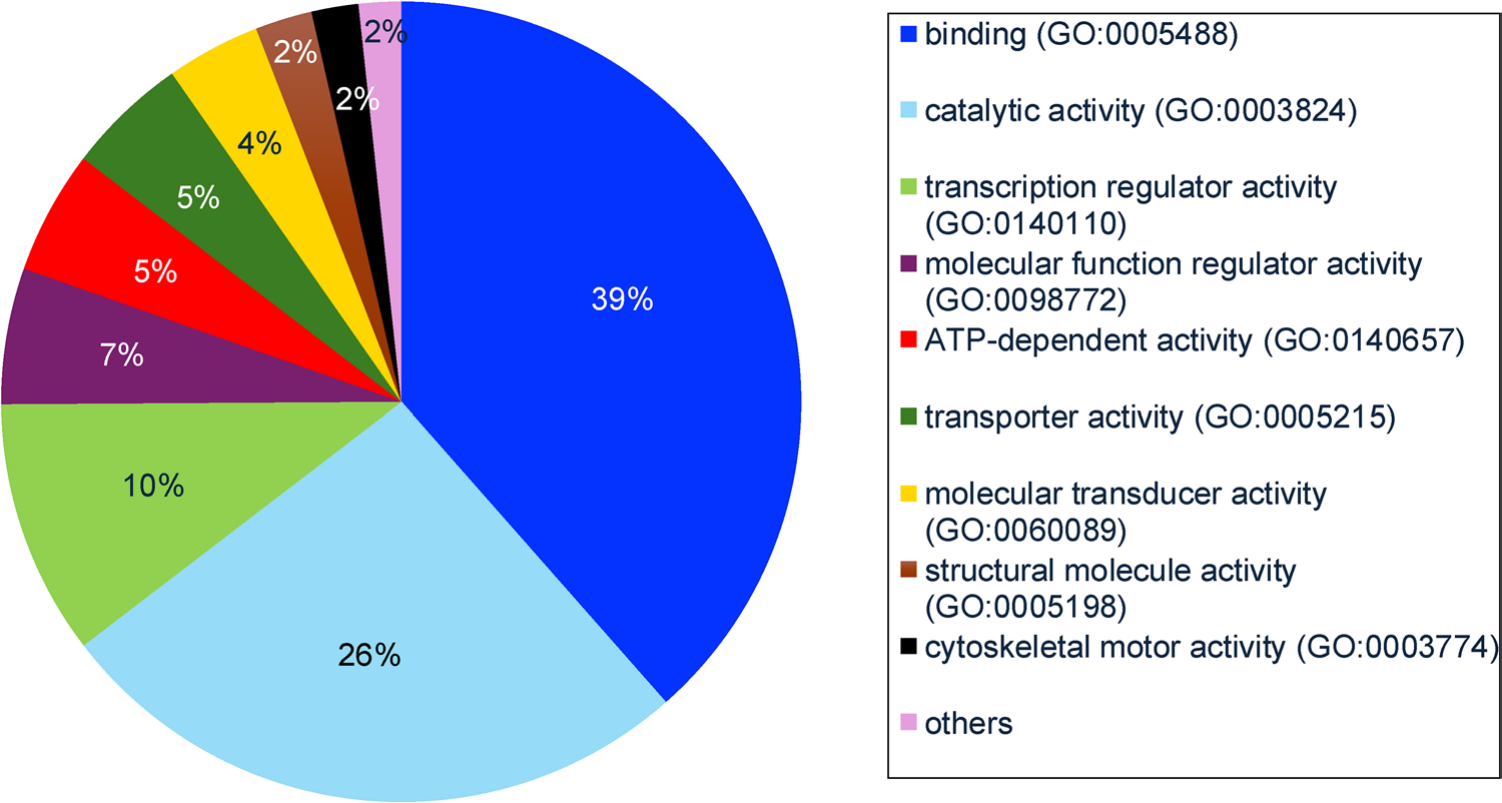
Functional classification of proteins found in deciduous teeth dental pulp according to GO molecular function terms

**Fig. 5:**
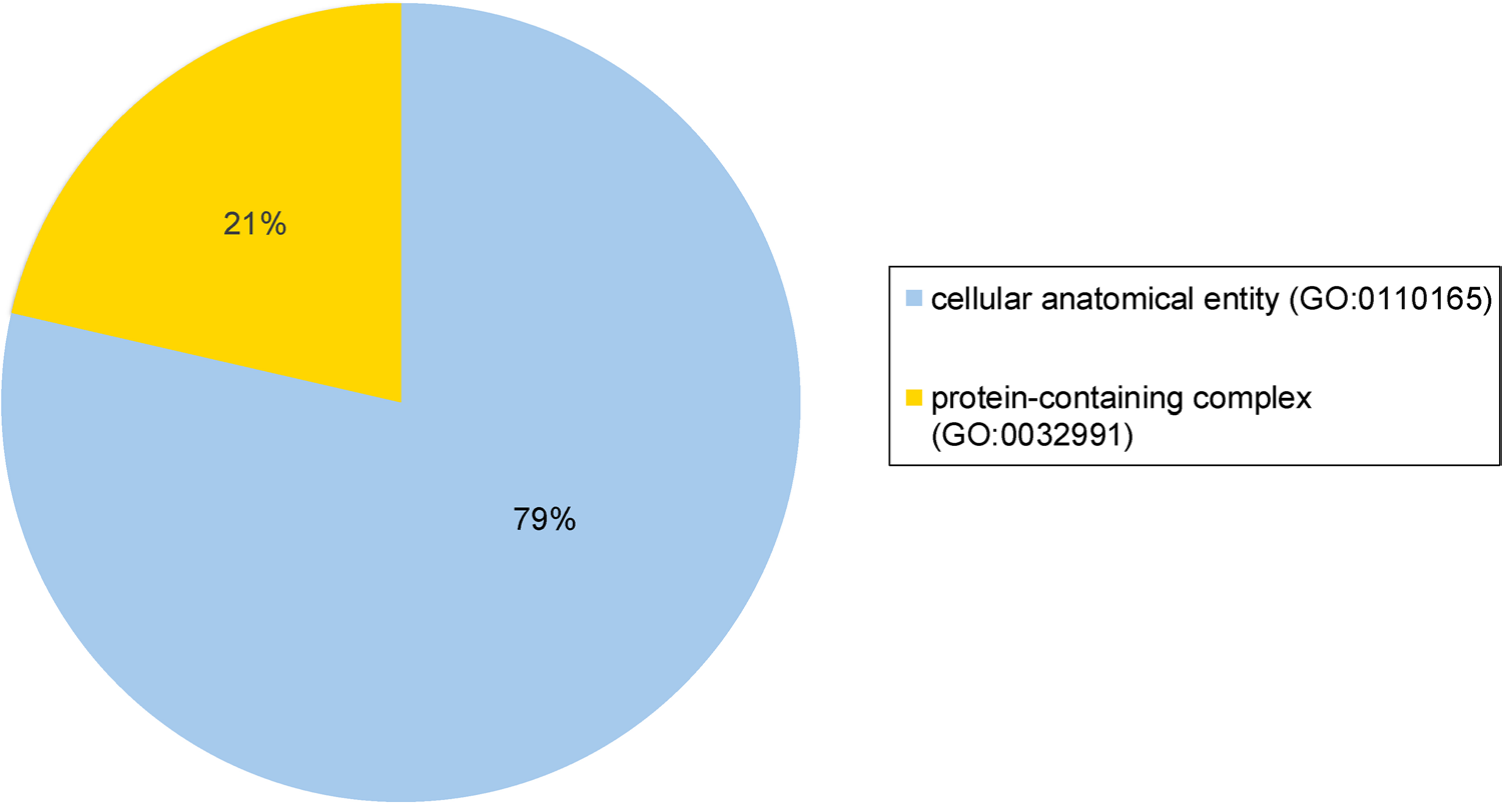
Functional classification of proteins found in deciduous teeth dental pulp according to GO cellular component terms

Dental pulp tissue samples were obtained from six different individuals, four girls and two boys aged 6 to 11 years (mean age: 8.86 years). The tooth samples comprised upper and lower deciduous anterior teeth (*n* = 6). In total, 3636 proteins were identified in dental pulp from these deciduous teeth. Details of proteins found in the pulp tissue of deciduous teeth were reported in Supplementary Table 1. GO enrichment analysis results indicating the general functional classification of proteins expressed in deciduous teeth pulp are presented in Figs. 3, 4, and 5, respectively, illustrating biological processes, molecular functions, and cellular components.

**Fig. 6:**
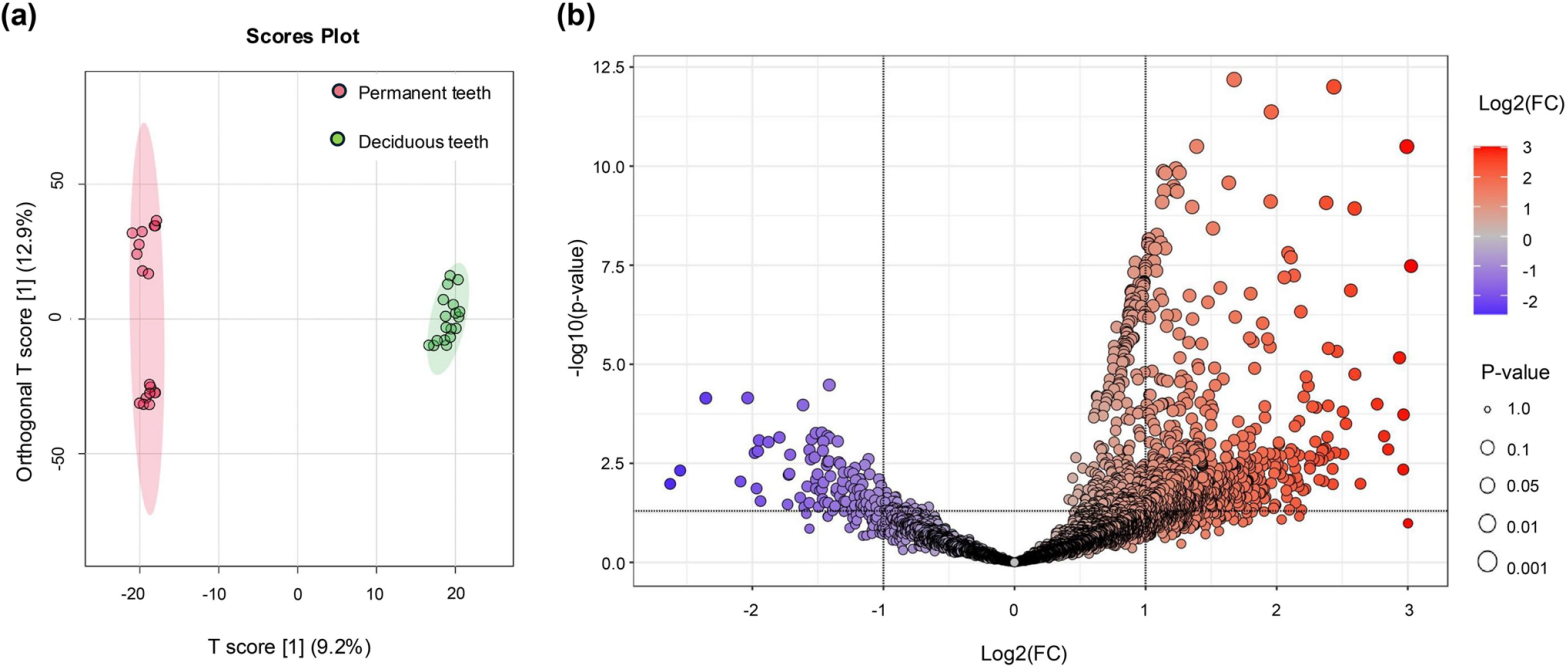
Comparison of the dental pulp proteomes of different dentitions. **a**: OPLS-DA distribution and clustering of protein samples between dentitions and within replicates. **b**: Volcano plot analysis of DEPs (*P*-value < 0.05, fold-change > 2) between the two dentitions. Red and bluish-purple colour, respectively, indicates high and low protein expression in the pulp of deciduous teeth

The obtained data for deciduous teeth were then compared with published data from permanent teeth previously deposited in the jPOST Repository (accession: PDX050860), which used the exact same protocols for protein extraction, quantification and mass spectrometry analysis (Lertruangpanya et al. 2024). Orthogonal partial least squares discriminant analysis (OPLS-DA) was applied to determine the distribution and clustering of samples among groups and within replicates, illustrated in Fig. 6a. Analysis of significantly different protein expression identified 736 DEPs between dental pulp of deciduous and permanent teeth, which comprised 643 proteins up-regulated and 93 down-regulated in deciduous dental pulp (Fig. 6b and Supplementary Table 2).

**Fig. 7:**
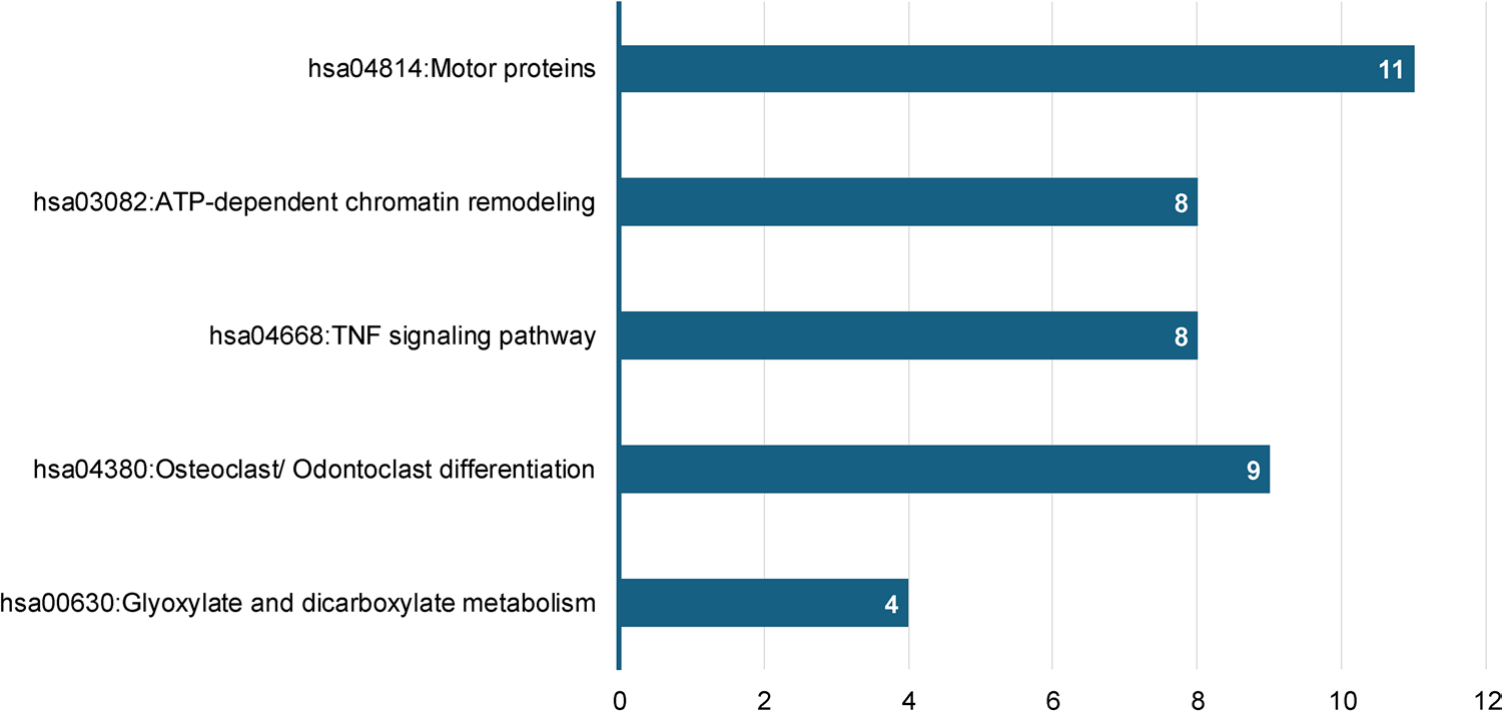
KEGG pathway analysis of DEPs between dental pulp of deciduous and permanent teeth (*P*-value < 0.1). Numbers in columns indicate the number of proteins involved in each pathway

**Fig. 8:**
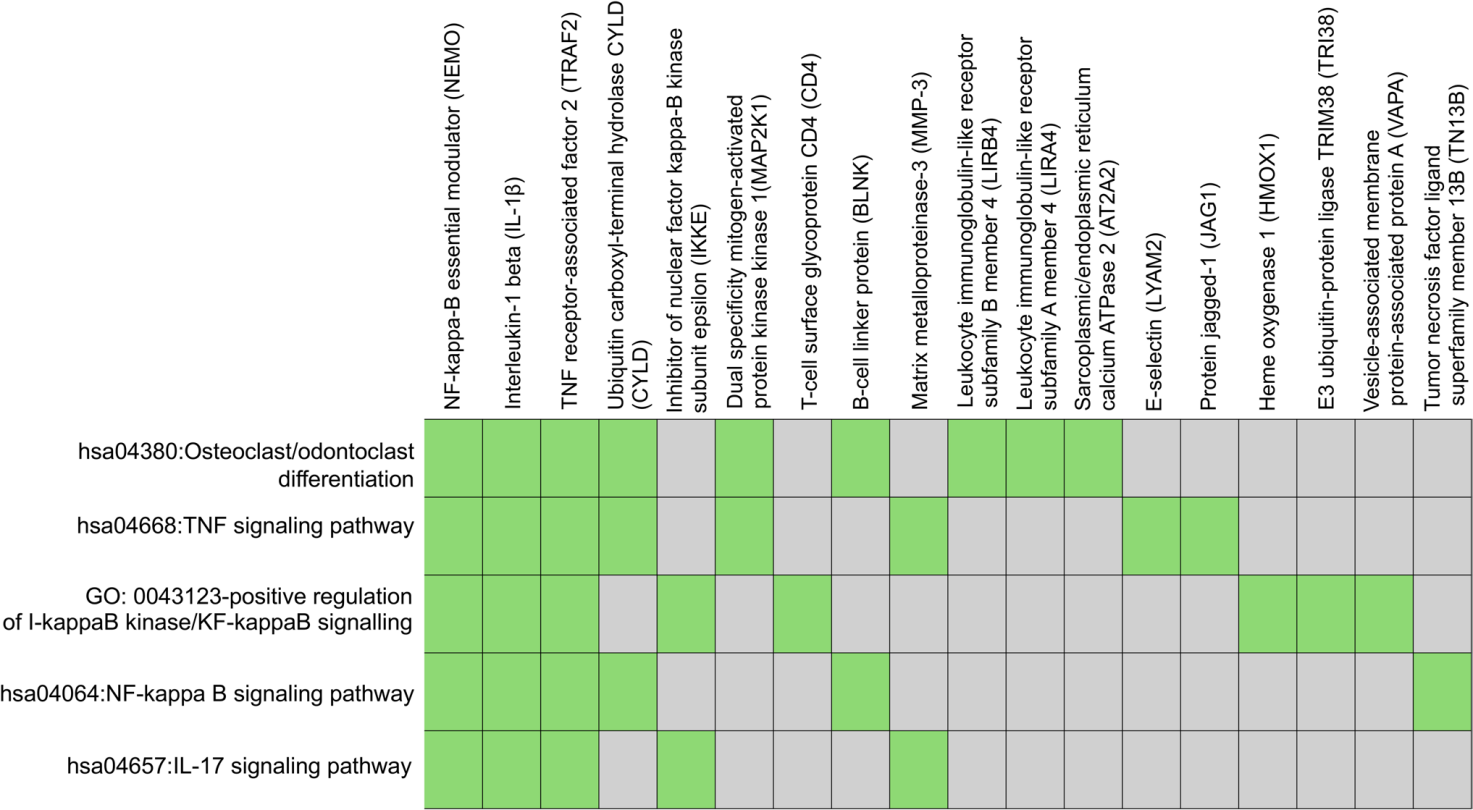
Mapping of DEPs to the annotation cluster enrichment analysis

**Fig. 9:**
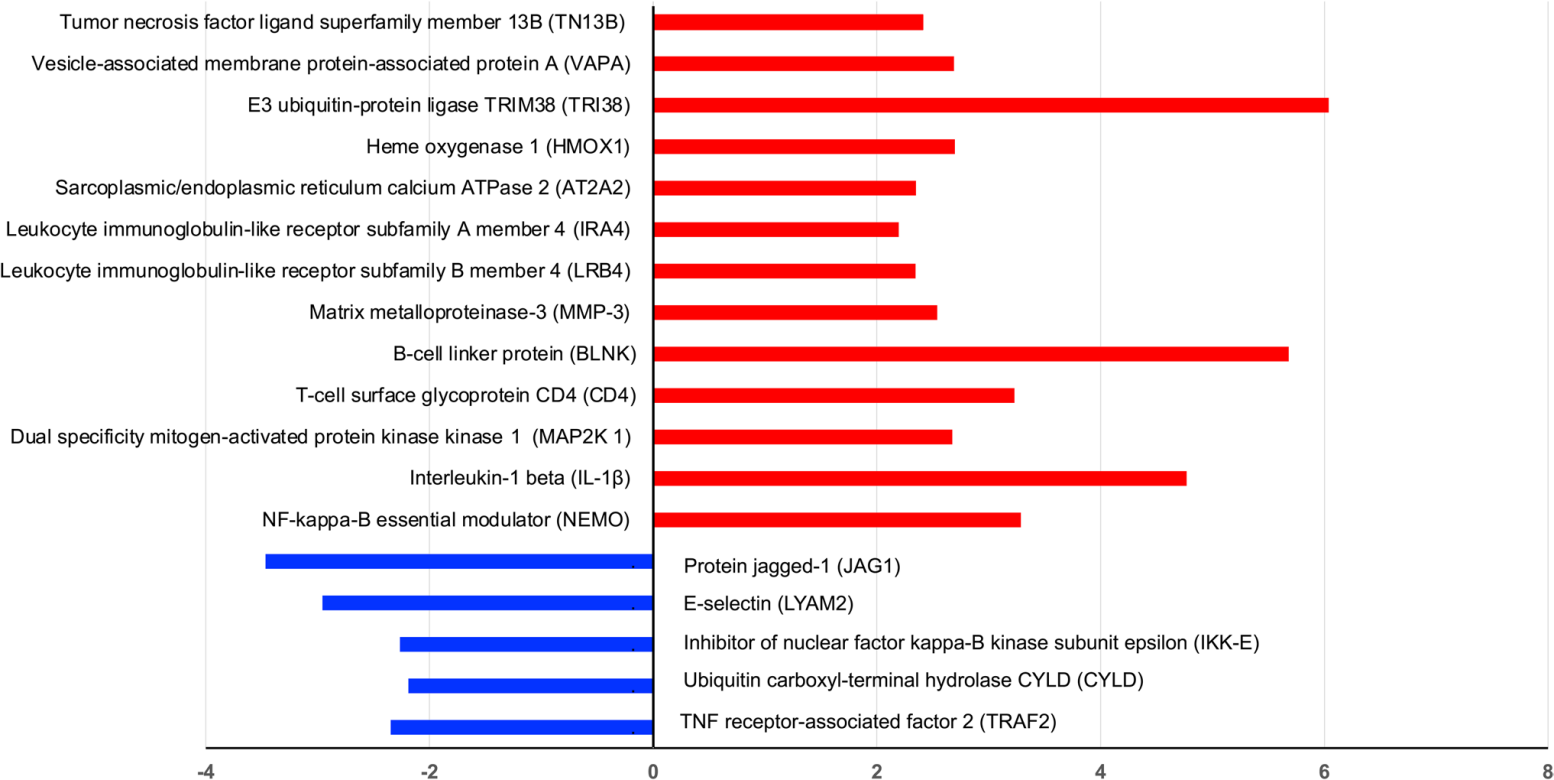
Bar chart representing fold changes in protein expression between deciduous and permanent dental pulp. Red and blue colour, respectively, indicates high and low protein expression in the pulp of deciduous teeth

KEGG pathway analysis of the DEPs revealed these proteins to include motor proteins and be involved in adenosine triphosphate (ATP)-dependent chromatin remodeling, tumor necrosis factor (TNF) signaling, osteoclast/odontoclast differentiation and glyoxylate and dicarboxylate metabolism (Fig. 7). We further mapped the DEPs to the annotation cluster enrichment analysis, which highlights collections of terms or pathways that have similar biological meanings on account of sharing similar proteins (Fig. 8). The expression of proteins from the annotation cluster enrichment analysis is shown in Fig. 9.

## Discussion

Teeth are ectodermal organs formed through a reciprocal relationship between the oral epithelium and the mesenchyme (Puthiyaveetil et al. 2016). Even though, in the grand scheme of things, deciduous and permanent teeth are organs that function in the same manner, many differences have been observed between them, including in the dental pulp (Arnold et al. 2019; Bardellini et al. 2016; Nukaeow et al. 2022).

Despite several studies having been conducted on the pulp proteome of permanent teeth, the corresponding proteome of deciduous teeth has not been investigated prior to this study. Analysis of biological process term enrichment showed proteins expressed in the deciduous dental pulp to have general functional classifications of cellular process, biological regulation, metabolic process, response to stimulus, localisation, and developmental process. These are similar to the terms identified for the dental pulp proteome of permanent teeth, as reported in previous studies (Eckhard et al. 2015; Eckhardt et al. 2014; Feridouni Khamaneh et al. 2021). Regarding molecular function terms, the top five functions in the dental pulp of deciduous teeth were binding, catalytic activity, transcription regulator activity, molecular function regulator activity and ATP-dependent activity. These likewise showed similarity with permanent dental pulp (Eckhard et al. 2015; Eckhardt et al. 2014; Feridouni Khamaneh et al. 2021), except for transcription regulator activity and ATP-dependent activity, which were more highly represented in deciduous dental pulp. Analysis of cellular component terms found the pulp proteome of deciduous teeth to be largely associated with cellular anatomical entity and protein-containing complex components also reported for the pulp proteome of permanent teeth (Eckhard et al. 2015; Eckhardt et al. 2014; Feridouni Khamaneh et al. 2021).

Despite these categorical similarities, OPLS-DA of pulp proteomes revealed the two dentitions to have completely separate clustering and discriminating characteristics, indicative of significant differences between them. Moreover, the statistical analysis identified 736 DEPs between deciduous and permanent teeth, with the majority being up-regulated in the dental pulp of deciduous teeth. KEGG pathway analysis and cluster enrichment analysis were performed to identify and map DEPs to signalling pathways and revealed the DEPs to be involved in TNF signalling, nuclear factor kappa B (NF-κB) signalling, and odontoclast/osteoclast differentiation. As with the total DEP set, most pathway-associated DEPs were highly expressed in deciduous dental pulp.

TNF signalling activation can promote cell differentiation, proliferation and apoptosis, and thereby ultimately lead to cell death or survival (Aggarwal 2003; Holbrook 2019). The TNF signalling pathway is additionally crucial in the immune response and closely related to the process of inflammation (Van Loo and Bertrand 2023). TNF-superfamily members primarily activate NF-κB, JUN N-terminal kinase, p38 mitogen-activated protein kinase (MAPK) and extracellular signal-regulated kinases (ERK1/ERK2) (Aggarwal 2003). According to the pathway analysis of DEPs, the main avenue of TNF signal transduction in pulp tissue may involve the NF-κB and MAPK pathways. In particular, dual-specificity mitogen-activated protein kinase kinase 1 (MAP2K1), an essential component for transduction and activation of MAPK signalling cascades (Avruch 2007), was up-regulated in the dental pulp of deciduous teeth. Likewise, matrix metalloproteinase-3 (MMP-3), an enzyme that degrades extracellular matrix components and is a well-known inflammatory mediator (Wan et al. 2021), was discovered to be up-regulated in the deciduous dental pulp. Increased MMP-3 production has been reported to be associated with TNF-α signalling activation of the NF-κB and p38-MAPK pathways in cementoblasts, potentially leading to the destruction and remodelling of periodontal tissues (Sanchavanakit et al. 2015).

The NF-κB signalling pathway is a well-established pathway known for regulating immune and inflammatory responses through the activation of a variety of genes involved in the inflammatory process (Lawrence 2009). Regulation of NF-κB signalling begins with the binding of pro-inflammatory cytokines, such as TNF-α or interleukin-1β (IL-1β), to their associated receptors, which initiates the downstream activation of the pathway (Chen and Chen 2013; Lawrence 2009). Such downstream signalling requires activation of the inhibitor of NF-κB kinase (IKK) complex, which consists of the IKKα subunit, IKKβ subunit and NF-κB essential modulator (NEMO). Activation of the IKK complex then leads to degradation of the inhibitor of NF-κB (IκB) and release of NF-κB molecules (Lawrence 2009; Solt et al. 2009). Downstream activation of the NF-κB pathway by IL-1β stimulation could also involve activation of ubiquitin E3-protein ligase, which eventually results in phosphorylation of IKKβ, leading to NF-κB activation (Chen and Chen 2013). In this study, proteins associated with NF-κB signalling, namely IL-1β, the E3 ubiquitin-protein ligase TRIM38 (TRI38) and NEMO, were found to be highly expressed in the dental pulp of deciduous teeth. Meanwhile, ubiquitin carboxyl-terminal hydrolase (CYLD), a tumour suppressor and deubiquitinating enzyme that inhibits NF-κB activity (Leeman and Gilmore 2008), was concurrently downregulated in the pulp of deciduous teeth.

These findings suggested that both the TNF and NF-κB signaling pathways are activated in the pulp of deciduous teeth. This could imply that the pulp of deciduous teeth is more prone to inflammatory and immune responses than that of permanent teeth, which might help to explain why some pulp therapy outcomes differ between deciduous and permanent teeth (Akhlaghi and Khademi 2015; Sanusi and Al-Bataynehb 2023; Silva et al. 2019).

Physiologic root resorption is a phenomenon unique to the deciduous dentition. The roots of deciduous teeth are only complete for a short period of time; resorption begins within three years after the roots are fully formed and continues throughout the tooth's life (Sheid and Weiss 2012). Even in the absence of a permanent successor, physiologic resorption of deciduous teeth proceeds, but at a relatively slow rate (Harokopakis-Hajishengallis 2007). Animal model research has revealed physiologic root resorption to be a complex early process involving a series of biological events, not just a single event that occurs prior to exfoliation (Lin et al. 2012; Murthy and Bhojraj 2023; Sasaki et al. 1990). In the interest of investigating the protein profiles and gaining a better understanding of pulp functions in deciduous dentition, the teeth used in this study were in the early stages of root resorption (Murthy et al. 2020), with resorption visible only in the apical third of the root.

Odontoclasts and osteoclasts are the cells responsible for root resorption (Harokopakis-Hajishengallis 2007). As one might expect, the enrichment analysis in this study identified odontoclast/osteoclast differentiation as an enriched pathway, with the majority of associated DEPs being up-regulated in the dental pulp of deciduous teeth. It has been confirmed that the functional characteristics, cellular mechanism, enzymatic properties, and differentiation process of odontoclasts are similar to those of osteoclasts, and the two cell types are believed to arise from the same origin (Harokopakis-Hajishengallis 2007; Kamat et al. 2013; Oshiro et al. 2001). TNF and NF-κB signalling are important in the activation of odontoblast/osteoclast differentiation (Harokopakis-Hajishengallis 2007; Kamat et al. 2013; Xiao et al. 2022); in addition, IL-1β, which was found to be highly expressed in the pulp of deciduous teeth, can also directly activate odontoblast/osteoclast differentiation (Harokopakis-Hajishengallis 2007; Kamat et al. 2013).

The activation of TNF and NF-κB inflammatory pathways, along with the differentiation of odontoblasts/osteoclasts in the pulp tissue of deciduous teeth, may shed light on why vital pulp therapy in deciduous teeth, particularly direct pulp capping and pulpotomy with calcium hydroxide, tend to result in internal resorption of deciduous dental pulp, leading to pulp treatment failure (Canoğlu et al. 2022; Jha et al. 2021; Moretti et al. 2008). In contrast, the same approaches have proven to be more effective in permanent teeth, resulting in the formation of a mineralised tissue barrier, and are, therefore, regarded as appropriate for vital pulp therapy in permanent teeth. (Akhlaghi and Khademi 2015; Ricucci et al. 2023).

The findings of this study indicated that there were considerable differences between the dental pulp of the two dentitions, with the pathway of inflammation and hard tissue resorption being particularly active in the pulp of deciduous teeth. Because of these differences, dental practitioners should be aware that even with identical dental procedures, the pulp of deciduous teeth may not respond in the same manner as permanent teeth, potentially leading to differing treatment outcomes. Particularly when performing dental procedures or utilising materials that are prone to triggering inflammatory reactions or odontoblast/osteoclast differentiation, it may result in a failure outcome in deciduous teeth. Understanding these characteristics could aid in determining an appropriate pulp therapy approach for deciduous teeth.

This study has some limitations with regard to donor group age difference, as deciduous teeth are usually extracted during middle or late childhood for reasons of prolonged retention or orthodontic purposes, whereas third molars are typically removed during late adolescence or adulthood. Furthermore, due to the timing of tooth removal, obtaining deciduous and permanent dental pulp from the same person is impractical. The sample size in this study is quite small because most deciduous teeth can naturally be exfoliated; thus, it is difficult to obtain deciduous teeth for which the majority of the root structure is still intact, in this case defined as having more than two-thirds of the root. Nonetheless, the data is sufficient to provide an initial screening of deciduous teeth pulp protein profiles, as well as statistical analysis and comparison of proteins from deciduous and permanent dental pulp. To investigate and expand on these findings, further research should be conducted with a larger sample size and should validate the proteins found to be differentially expressed between dental pulp of different dentitions.

## Conclusion

This is the first study to identify and compare the proteomic profiles of deciduous pulp to those of permanent pulp. Within its limitations, it has been shown that although the overall characteristics of dental pulp were generally similar for both dentitions, there were notable and significant differences in terms of individual protein expression. As a result, despite using the same treatment approach, the pulp of deciduous teeth may react differently than that of permanent teeth, as in cases where clinical procedures known to induce inflammation or hard tissue resorption result more frequently in unfavourable outcomes in deciduous teeth.

## Supplementary Information

Below is the link to the electronic supplementary material.

Supplementary file1 (XLSX 193 KB)

## Data Availability

Data are provided within the manuscript or supplementary information and the raw mass-spectrometric data were deposited in jPOST Repository https://repository.jpostdb.org (accession: PDX052858).
